# Intrahepatic cholangiocarcinoma induced M2-polarized tumor-associated macrophages facilitate tumor growth and invasiveness

**DOI:** 10.1186/s12935-020-01687-w

**Published:** 2020-12-07

**Authors:** Hui Yuan, Zelong Lin, Yingjun Liu, Yuchuan Jiang, Ke Liu, Mengxian Tu, Nan Yao, Chen Qu, Jian Hong

**Affiliations:** 1 Department of Pathophysiology, School of Medicine, Jinan University, Guangzhou, 510630 Guangdong China; 2 Department of Gastroenterology, Huizhou Municipal Central Hospital, Huizhou, 516001 Guangdong China; 3 Cancer Center, Integrated Hospital of Traditional Chinese Medicine, Southern Medical University, Guangzhou, 510315 Guangdong China; 4 Department of General Surgery, Affiliated Tumor Hospital of Zhengzhou University, Zhengzhou, 450008 Henan China; 5 College of Pharmacy, Jinan University, Guangzhou, 510632 Guangdong China; 6 Department of Oncology, The Second Affiliated Hospital of Guangzhou Medical University, Guangzhou, 510260 Guangdong China

**Keywords:** Intrahepatic cholangiocarcinoma, M2 macrophage, Signal transducer and activator of transcription 3 (STAT3), Epithelial–mesenchymal transition

## Abstract

**Background:**

M2-polarized tumor-associated macrophages (M2-TAMs) have been shown to correlate with the progression of various cancers, including intrahepatic cholangiocarcinoma (ICC). However, the interactions and mechanism between M2 macrophages and ICC are not completely clear. We aimed to clarify whether M2 macrophages promote the malignancy of ICC and its mechanism.

**Methods:**

Two progressive murine models of ICC were used to evaluate the alterations in different macrophage populations and phenotypes. Furthermore, we assessed M2 macrophage infiltration in 48 human ICC and 15 normal liver samples. The protumor functions and the underlying molecular mechanisms of M2 macrophages in ICC were investigated in an in vitro coculture system.

**Results:**

We found that the number of M2 macrophages was significantly higher in ICC tissues than in normal bile ducts in the two murine models. M2 macrophage infiltration was highly increased in peritumoral compared with intratumoral regions and normal liver (*p* < 0.01). ICC cells induced macrophages to differentiate into the M2-TAM phenotype, and coculture with these M2 macrophages promoted ICC cell proliferation, invasion and epithelial–mesenchymal transition (EMT) in vitro. Mechanistically, M2-TAM-derived IL-10 promoted the malignant properties of ICC cells through STAT3 signaling. Furthermore, blockade of IL-10/STAT3 signaling partly rescued the effects of M2 macrophages on ICC.

**Conclusion:**

Our results indicated that M2-polarized macrophages induced by ICC promote tumor growth and invasiveness through IL-10/STAT3-induced EMT and might be a potential therapeutic target for ICC.

## Background

Intrahepatic cholangiocarcinoma (ICC) is an epithelial cell malignancy and the second most common primary liver cancer, constituting approximately 10–15% of all primary liver malignancies [1, 2]. The incidence and mortality rates of ICC have been rising in recent years, and the highly malignant and invasive characteristics result in a poor prognosis of ICC patients, with a 5 year survival rate of only 20–40% after surgery [3, 4].

Chronic inflammation and injury of bile duct cells are known ICC-promoting conditions [5]. During cancer-related chronic inflammation, leukocytes, such as macrophages, dendritic cells, mast cells and T cells, are recruited to the tumor microenvironment to play a role in oncogenesis [6]. Among them, tumor-associated macrophages (TAMs) are the most frequently encountered stromal cells in the tumor microenvironment and are alternatively activated [7, 8], promoting cancer proliferation, epithelial-mesenchymal transition (EMT), invasion and metastasis [9, 10], which play an important role in modulating tumor growth and progression [11, 12]. Moreover, cancer cells can activate macrophages and consequently induce tumor malignancy. This suggests a positive feedback between tumor cells and TAMs to promote malignancy [13].

As is reported, tumor-derived factors induce the differentiation of macrophages into the M2 phenotype, and M2-TAM-conditioned medium can mediate ICC cell migration with increasing N-cadherin expression, but only in vitro. The biological function and regulatory mechanism of M2 macrophages in ICC remain elusive, and there is no evidence to illustrate their role in ICC in vivo [14, 15].

In this study, to further understand the interactions that take place between ICC cells and M2 macrophages and how they contribute to the malignancy of ICC in vitro and in vivo, we first generated two progressive murine models of ICC, a thioacetamide rat model and diethylnitrosamine-left median bile duct ligation (LMBDL) mouse model, to evaluate the alteration of different macrophage populations and phenotypes during ICC progression [16, 17]. We then induced M2 macrophage polarization in vitro from monocyte THP-1 cells and cocultured them with ICC cells to investigate whether M2 macrophages could promote ICC cell proliferation, invasion and migration.

## Materials and methods

### Patient samples

Human liver tissues, which included tumorous tissues (intratumor) and corresponding paracarcinoma tissues (peritumor), were collected from 48 patients who underwent radical resection for ICC at the Affiliated Tumor Hospital of Zhengzhou University in 2018. In addition, 15 normal liver tissue samples were obtained from hemangioma during the same period.

### Cell culture

Human ICC cell lines (HuCCT1, Huh28) and THP-1 cells were obtained from American Type Culture Collection (ATCC) and were cultured in RPMI-1640 medium (Gibco, USA) supplemented with 10% fetal bovine serum (FBS, Gibco) at 37 °C with a humidified atmosphere of 5% CO_2_.

### M2 polarized macrophage induction

To obtain M2-polarized macrophages, we induced THP-1 monocytes in vitro [18]. THP-1 cells were cultured in RPMI-1640 supplemented with 200 nM phorbol myristate acetate (PMA, Peprotech, USA) for 6 h to produce M0 macrophages, and then 20 ng/ml recombinant interleukin 4 (IL-4, Peprotech) and recombinant interleukin 13 (IL-13, Peprotech) were added for an additional 18 h to induce M2-polarized macrophages.

### Animal studies

All rats and mice were purchased from Guangdong Medical Laboratory Animal Center. The animal experimental protocol was reviewed and approved by the Institutional Animal Ethical Committee, Experimental Animal Center of Southern Medical University, and followed the Guide for the Care and Use of Laboratory Animals by the US National Institutes of Health.

#### The thioacetamide model of ICC

The thioacetamide (TAA) rat model of cholangiocarcinoma was established as reported [16]. TAA (Sigma, USA) was given in drinking water at a standard dose of 0.03% for male Sprague–Dawley (SD) rats (350 ± 20 g). Foci of cholangiocyte proliferation could be observed at the 9th week, and dysplasia could be detected at the 12th week. Whitish and visible ICC tumors could be observed at the 16th week after administration. All animals developed multiple different sized invasive tumors at 24 weeks.

#### The DEN-left median bile duct ligation model of ICC

The construction of this model was performed as reported [17]. To achieve tumor development in mice, we subjected 7 week-old male BALB/c mice to two separate weekly intraperitoneal injections of 100 mg/kg diethylnitrosamine (DEN, Sigma). After 2 weeks, left median bile duct ligation was performed in all experimental mice. After a week, DEN (25 mg/kg) was administered by oral gavage once a week, and the total duration of the experiment lasted 28 weeks, with ICC formation.

#### Subcutaneous xenograft experiments

Huh28 cells (2 × 10^6^) were cocultured with 1 × 10^6^ M2 macrophages (THP-1-induced) or monocytes (THP-1) as a control, and then the cell mixtures were subcutaneously injected into the flanks of 5 week-old male BALB/c nude mice (n = 8/group). Tumor size was measured by Vernier calipers, and the tumor volume was calculated as length × (width)^2^ × 0.5. Mice were sacrificed at day 15 after injection, and tumors were excised, weighed, and processed for analysis.

### Cell proliferation assay

The cell proliferation ability was determined using the Cell Counting Kit-8 (CCK8, Dojindo, Japan). Briefly, 3000 cells were seeded into 96-well plates (Corning, USA). At the indicated times, each well medium was replaced with an equal volume of fresh medium containing 10% CCK-8 reagent. After cells were incubated at 37 °C for 2.5 h, absorbance was measured using an Enzyme-linked immunosorbent instrument (Thermo-Fisher, USA) at 450 nm. Recombinant human interleukin 10 (rhIL-10, R&D Systems, USA) and its neutralizing antibody (Anti-IL-10, Abcam, UK) were used in this study.

### Macrophage and ICC cell coculture

ICC cell and THP-1/M2 macrophage coculture was conducted using 0.4-μm diameter transwell chambers (Corning, USA). A total of 1 × 10^5^ M2 macrophages polarized from THP-1 cells were seeded in the upper chamber, and then inserts containing THP-1 or M2 macrophages were placed into 6-well cell culture plates seeded with HuCCT1 or Huh28 cells (1 × 10^5^ cells per well) in advance and incubated for up to 72 h. Coculture systems were treated with rhIL-10 or its neutralizing antibody or the STAT3 inhibitor, S3I-201 (Selleck Chemicals, USA). Cells in the upper and lower chambers were collected and used for qRT-PCR or Western blotting to detect changes in M2-related gene expression in macrophages and STAT3 or its phosphorylation and epithelial-mesenchymal transition (EMT)-related genes in ICC cells.

### Invasion and migration assays

Transwell chambers 8.0 μm in diameter (Corning, USA) were used to evaluate cell invasion and migration. For the invasion assay, the upper chamber was coated with 60 μl of a mixture of Matrigel (BD Biosciences, USA) and RPMI-1640 without serum (1:8) and allowed to solidify at 37 °C for 2 h. Then, 1.0 × 10^4^ cells were seeded into the upper chamber, and the bottom chamber was filled with 500 μl of RPMI-1640 supplemented with 10% FBS. After incubation at 37 °C for 36 h, cotton swabs were used to remove the Matrigel and the cells remaining in the upper chamber. For the migration assay, 1.0 × 10^4^ cells were seeded into the upper chamber with serum-free RPMI-1640. After incubation at 37 °C for 12 h, cotton swabs were used to remove the cells remaining in the upper chamber. The cells were counted and imaged under a light microscope. Each experiment was performed in triplicate.

### Immunohistochemistry (IHC) and immunofluorescence (IF)

Immunostaining was performed to determine the expression levels of F4/80, iNOS, CD163/CD206, Ki-67, E-cadherin, vimentin, p-STAT3 and IL-10 antibodies as reported. IHC staining and scoring were performed as described previously [19]. A biotinylated secondary antibody and diaminobenzidine (DAB) kit (Dako, Denmark) were used according to the manufacturer’s protocol. The density of positive IHC staining was measured with a computerized imaging system (Toupcam, China). The density was quantified by ImageJ software.

After coculture with THP-1-derived macrophages, ICC cells were fixed with 4% paraformaldehyde and permeabilized with 0.1% Triton X-100. Next, these cells were incubated with primary antibodies overnight at 4 °C, followed by incubation with fluorescein-labeled secondary antibody (1:1,000; Santa Cruz Biotechnology, USA) at room temperature for 1 h. After the final washes with phosphate-buffered saline, the samples were stained with DAPI and examined using a laser confocal microscope (Zeiss, Germany).

The primary antibodies used for IHC and IF are listed in Additional file 1: Table S1.

### ELISA assay

IL-10 levels were quantified in the supernatants of ICC cell (HuCCT1 and Huh28), monocyte and THP-1-derived M2 macrophage monocultures and ICC cell/THP-1 and ICC cell/M2 macrophage cocultures using an IL-10 Human ELISA kit from MEIMIAN (China). All procedures were performed according to the manufacturer’s instructions.

### Real-time quantitative PCR (qRT-PCR)

Total RNA was extracted from cell lines using TRIzol Reagent (Invitrogen, USA) and used to synthesize complementary DNA (cDNA) with a reverse transcriptase kit (Takara Bio Inc, Dalian, China) in accordance with the manufacturer’s instructions. Subsequently, qRT-PCR analysis was performed using SYBR® Green I Master Mix (Roche, Switzerland) on the LightCycler®480 platform. Primers used for the amplification of human genes are shown in Additional file 2: Table S2.

### Western blotting

Western blotting was performed as described previously [19]. Briefly, protein was extracted from cells using RIPA lysis buffer with protease/phosphatase inhibitor (Thermo-Fisher), separated by a NuPAGE Novex 4–12% Bis–Tris Gel (Invitrogen, USA) and then transferred to PVDF membranes. The PVDF membranes were blocked with 5% nonfat milk for 1.5 h at room temperature and then incubated with the corresponding primary antibodies overnight at 4 °C. Subsequently, the PVDF membranes were washed with TBST and incubated with the secondary antibodies. The corresponding band was revealed using enhanced chemiluminescence reagents (Pierce, Rockford, IL). The primary antibodies used for Western blotting are listed in Additional file 1: Table S1.

### Statistical analysis

All statistical analyses were performed with GraphPad Prism 7.00 software (San Diego, CA, USA). Quantitative data were analyzed by Student’s *t* test. At least three samples were tested in each assay. All data are represented as the means ± SD, and a *p* value < 0.05 was considered statistically significant.

## Results

### The number of M2 macrophage was increased in two murine models of ICC spontaneous induction

**Fig. 1:**
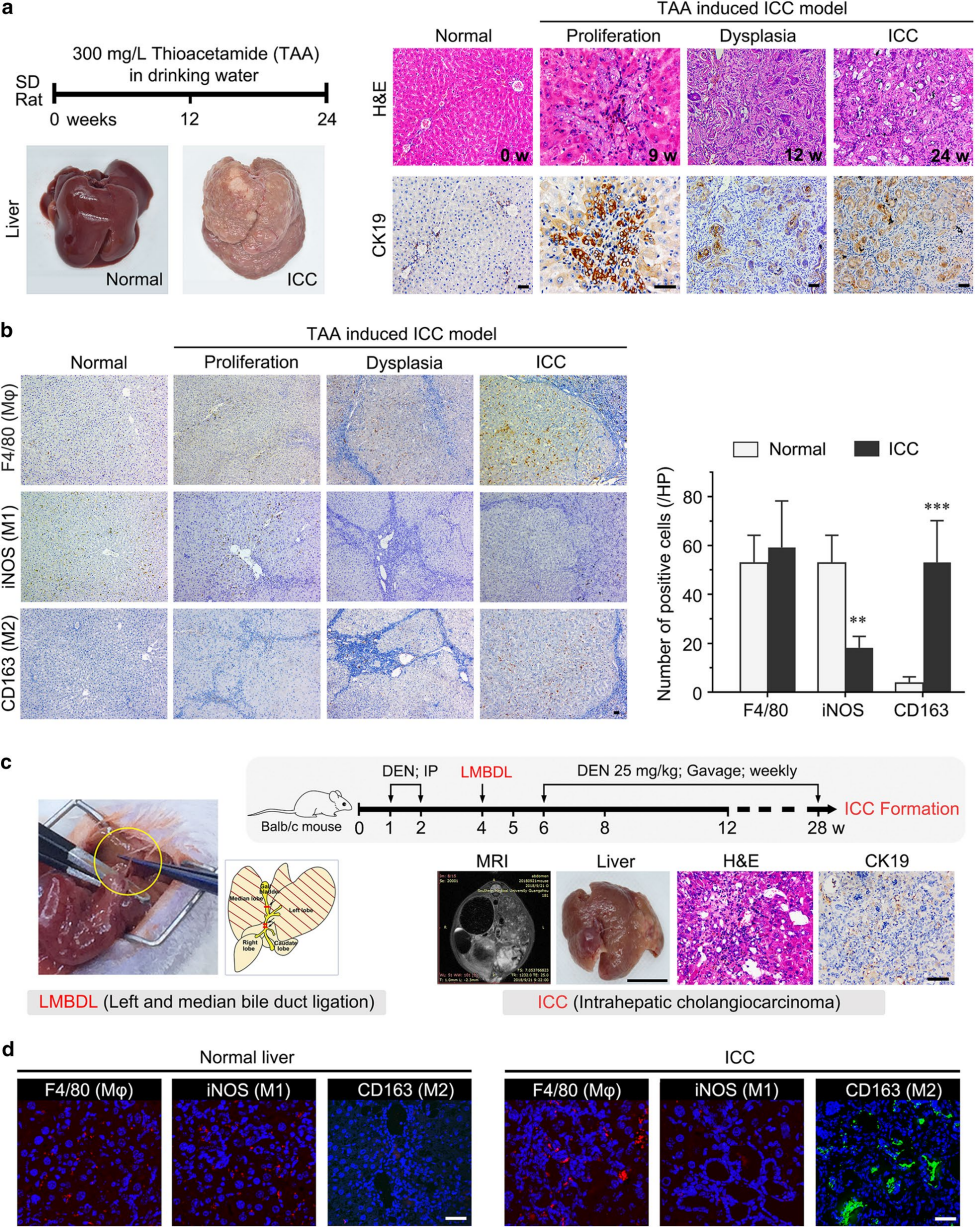
The number of M2 macrophage was increased in two murine models of ICC spontaneous induction. **a** Schematic of the experimental design of the thioacetamide rat model of ICC and representative images of rat liver stained with H&E and CK19. **b** The left panel shows representative images of F4/80, iNOS and CD163 immunohistochemistry staining of rat bile duct disease and normal tissues, and the right panel shows the analysis of IHC staining between ICC (n = 16) and normal tissues (n = 5). **c** Schematic of the experimental design of the DEN-left median bile duct ligation (LMBDL) model of ICC. The left panel shows a diagram of the LMBDL position, and the right panel shows representative images of mouse liver stained with H&E and CK19. **d** Immunofluorescence staining for monocytes/macrophages (F4/80), M1 macrophages (iNOS), and M2 macrophages (CD163) in mouse ICC and normal liver tissues (n = 3/group). The red/green signal represents individual marker staining, and the blue signal represents DAPI-stained nuclei. The data are presented as the mean ± SD; **p < 0.01, ***p < 0.001. Bar = 1 mm

To evaluate the expression of macrophages and its phenotypes, we successfully generated two murine spontaneous induction models of ICC (which develop as a consequence of biliary hyperplasia and atypical hyperplasia), including a thioacetamide rat model and diethylnitrosamine (DEN)-LMBDL mouse model (Fig. 1a, c). Three antibodies, targeting monocytes/macrophages (F4/80), M1 macrophages (iNOS), and M2 macrophages (CD163), were used to identify distinct subpopulations by immunofluorescence and immunohistochemistry. In the rat spontaneous induction model of ICC, we found that M2 macrophages were more abundantly present, while M1 macrophages were decreased in the stroma of ICC samples compared to normal tissues (*p* < 0.01). Furthermore, we did not observe an increase in M2 macrophages during bile duct hyperplasia and dysplasia (Fig. 1b). We also found a similar change in the BALB/c ICC mouse model, in which M2 macrophages were increased in the stroma of ICC samples compared to normal tissues (Fig. 1d).

### M2 macrophage infiltration is increased in human ICC tissues

**Fig. 2:**
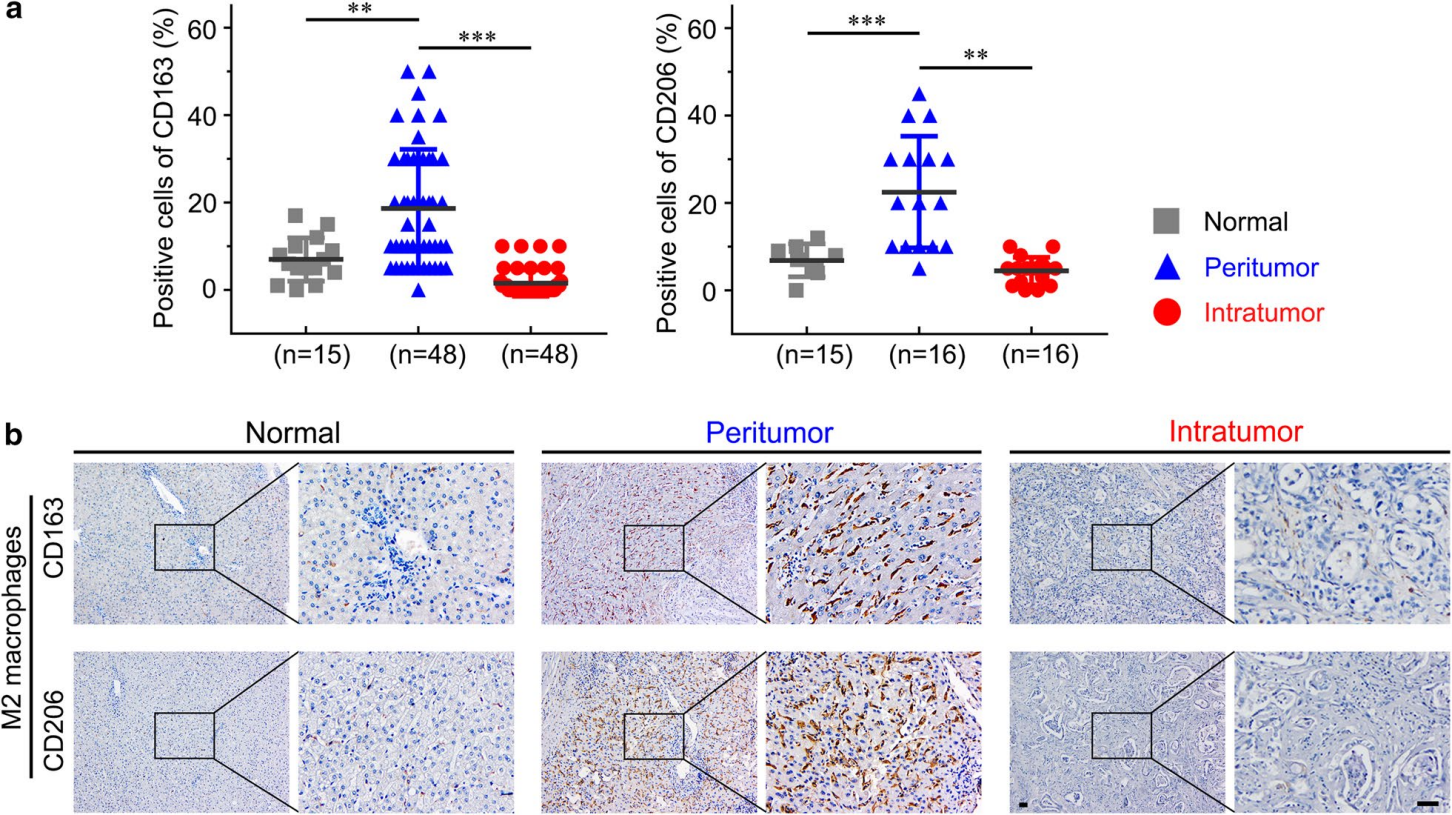
M2 macrophage infiltration is increased in human ICC tissues. **a** Analysis of CD163 + and CD206 + M2 macrophages infiltrating ICC and normal liver tissues. **b** Immunohistochemical staining of CD163 and CD206 (M2 macrophage markers) in normal liver and ICC tissues. The upper and lower panels represent images of tissues stained with anti-CD163 and anti-CD206 antibodies, respectively. The data are presented as the mean ± SD; **p < 0.01, ***p < 0.001. Bar = 1 mm

It has been reported that M1 macrophages have little effect on cancer cells [20]. In our study, we found that M1 macrophages were also decreased in the two murine ICC models. Thus, we focused more on M2 macrophages. We assessed the number of M2 macrophages in intratumoral and peritumoral tissue regions of 48 ICC samples and 15 normal liver tissue samples by immunohistochemistry. The results showed that both CD163 and CD206 were expressed at a higher level in the peritumoral region than in the intratumoral region and normal liver tissues (*p* < 0.01), which indicated that the number of M2 macrophage was elevated in the tumor peripheral area (Fig. 2a, b). It has been previously reported that M2 macrophages promote proliferation and invasiveness in various cancers, and we next investigated the role of M2 macrophage infiltration in the malignant properties of ICC.

### ICC cells induced M2 macrophage polarization

**Fig. 3:**
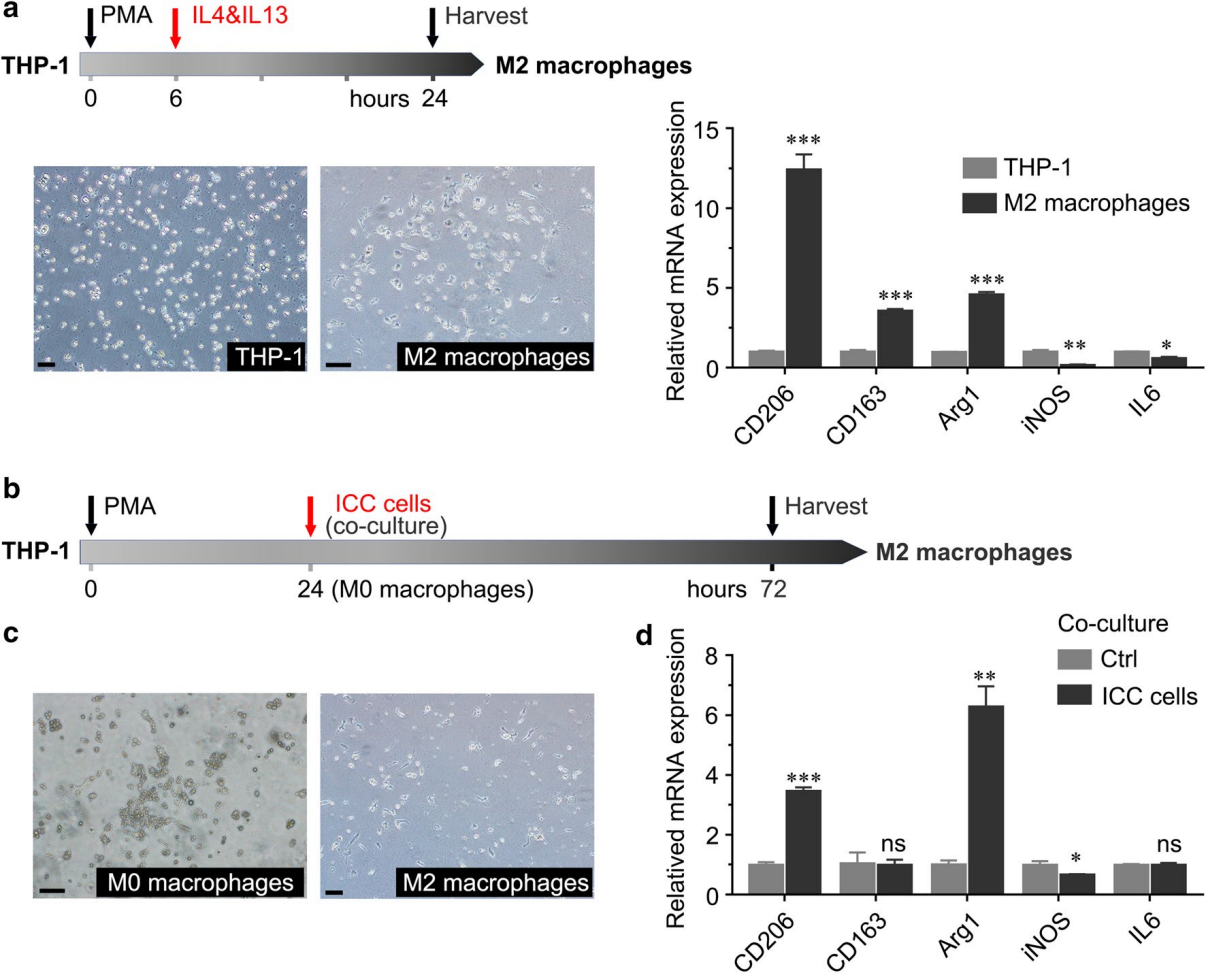
ICC cell-induced M2 macrophages polarized from monocytic THP-1 cells. **a** Schematic of published methods to induce M2-polarized macrophages from THP-1 cells. The left panel shows that THP-1 cells treated with PMA, IL-4 and IL-13 polarized into M2 macrophages. The relative expression of cytokines (CD206, CD163, Arg1, iNOS and IL-6) in M2 macrophages between THP-1 cells and M2 macrophages was analyzed by qRT-PCR. **b** Schematic of M2-like polarized macrophages induced by ICC cells. After treatment with 200 nM PMA for 24 h, THP-1 cells differentiated into unpolarized (M0) macrophages. M0 macrophages cocultured with ICC cells for 48 h polarized into M2-phenotype macrophages. **c** Representative images of M0 macrophages and M2 macrophages. **d** The relative expression of CD206, CD163, Arg1, iNOS and IL-6 in M0 macrophages after coculture with ICC cell lines was analyzed by qRT-PCR. The data are presented as the mean ± SD; *p < 0.05, **p < 0.01, ***p < 0.001. Bar = 1 mm

To demonstrate the interactions that take place between ICC cells and M2-polarized tumor-associated macrophages, we induced M2 macrophage polarization from monocytic THP-1 cells in vitro according to previously published methods [18]. M2 macrophages became adherent and exhibited pseudopod formation, in contrast to the suspended state of THP-1 cells (Fig. 3a). We found that the expression of the M2 macrophage markers CD206, CD163 and Arg1 was significantly increased compared to that in undifferentiated THP-1 cells, while there was a decrease in the mRNA expression of the M1 markers iNOS and IL-6 (Fig. 3a). THP-1 cells were pretreated with PMA at 200 nM for 24 h to obtain M0 macrophages. Then, they were cocultured with ICC cells for 48 h, and we observed that the number of cells extending pseudopods and the expression of CD206 and Arg1 were significantly increased compared to those in control cultures (Fig. 3b–d). Thus, ICC cells can induce tumor-associated macrophage differentiation into the M2 phenotype after PMA treatment.

### M2 macrophages promote tumor growth and invasiveness

**Fig. 4:**
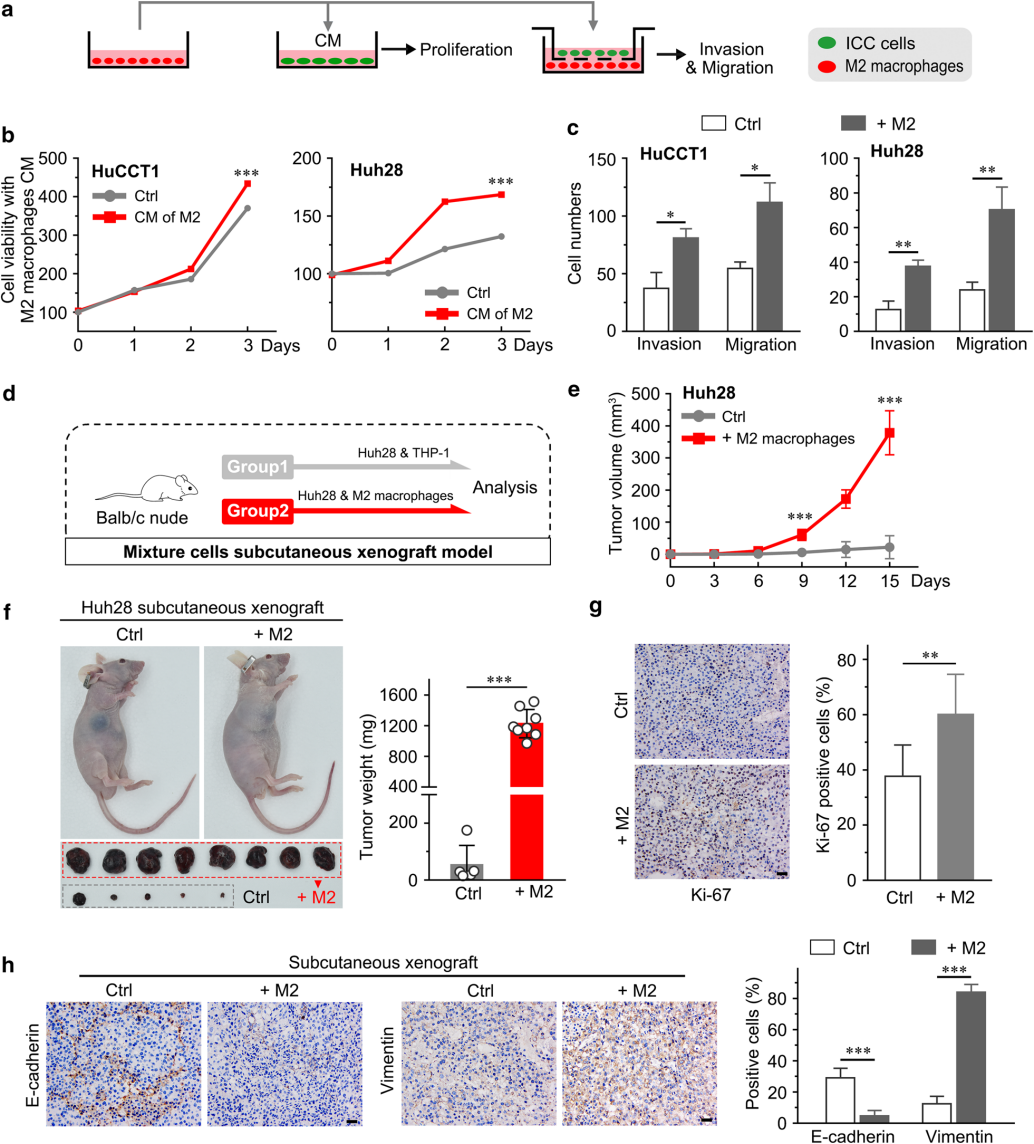
M2 macrophages promote tumor growth and invasiveness. **a** Experimental design to investigate ICC cell proliferation and invasiveness in the presence of M2 macrophages in vitro. **b** CCK8 assays show that M2 macrophages promoted proliferation in HuCCT1 and Huh28 cells. **c** Invasion and migration assays show that M2 macrophages promoted ICC cell invasiveness in coculture. **d** Experimental design to investigate the effects of M2 macrophages on ICC growth in the Huh28 and M2 macrophage co-implantation model. **e** Changes in subcutaneous tumor volume in the Huh28 and Huh28 + M2 groups over time. **f** Representative images of mice from the two groups, and subcutaneous tumor weights were measured after excision. **g** Representative images of Ki-67 IHC (left panel) and analysis (right panel). **h** Immunohistochemical (IHC) staining of E-cadherin and Vimentin performed on subcutaneous tumor sections. The data are presented as the mean ± SD; *p < 0.05, **p < 0.01, ***p < 0.001. *CM* culture supernatant

To investigate whether M2 macrophages could promote ICC cell proliferation, invasion and migration, we added the supernatant from M2 macrophages to ICC cells, or macrophages were cocultured with ICC cells in transwell chambers (Fig. 4a). We found that supernatant from M2 macrophages induced HuCCT1 and Huh28 cell proliferation in vitro (*p* < 0.001, Fig. 4b). The migration and invasion capacities of both HuCCT1 and Huh28 cells were significantly enhanced after coculture with M2 macrophages (*p* < 0.05, Fig. 4c).

Next, we investigated whether M2 macrophages could sustainably facilitate ICC growth in vivo (n = 8/group). Huh28 cells were cocultured with M2 macrophages or THP-1 cells and then subcutaneously co-injected into nude mice (Fig. 4d). As expected, Huh28 cells cocultured with M2 macrophages and implanted in nude mice generated larger tumors, and the tumor weight was greater than that of the negative control group (*p* < 0.001, Fig. 4e, f). Furthermore, we assessed Ki-67 expression in mouse xenografts by immunohistochemistry and found more positive cells in xenografts from the experimental group than in the control group (*p* < 0.01, Fig. 4g), suggesting that M2 macrophages significantly facilitate ICC tumor growth in vivo. In addition, the expression of EMT markers were significantly changed in the subcutaneously co-transplantation tumor (Fig. 4h). We found that vimentin expression was significantly upregulated, while E-cadherin expression was downregulated in the co-transplantation tumor tissues (*p* < 0.001, Fig. 4h). It has been well reported that EMT enhanced the invasion capacity of tumor cells [21]. These results were consistent with the in vitro data and suggested that the co-transplantation with M2 macrophages increased invasion capacity of ICC cells.

### IL-10 is responsible for the pro-proliferative and invasive effects of M2 macrophages on ICC

**Fig. 5:**
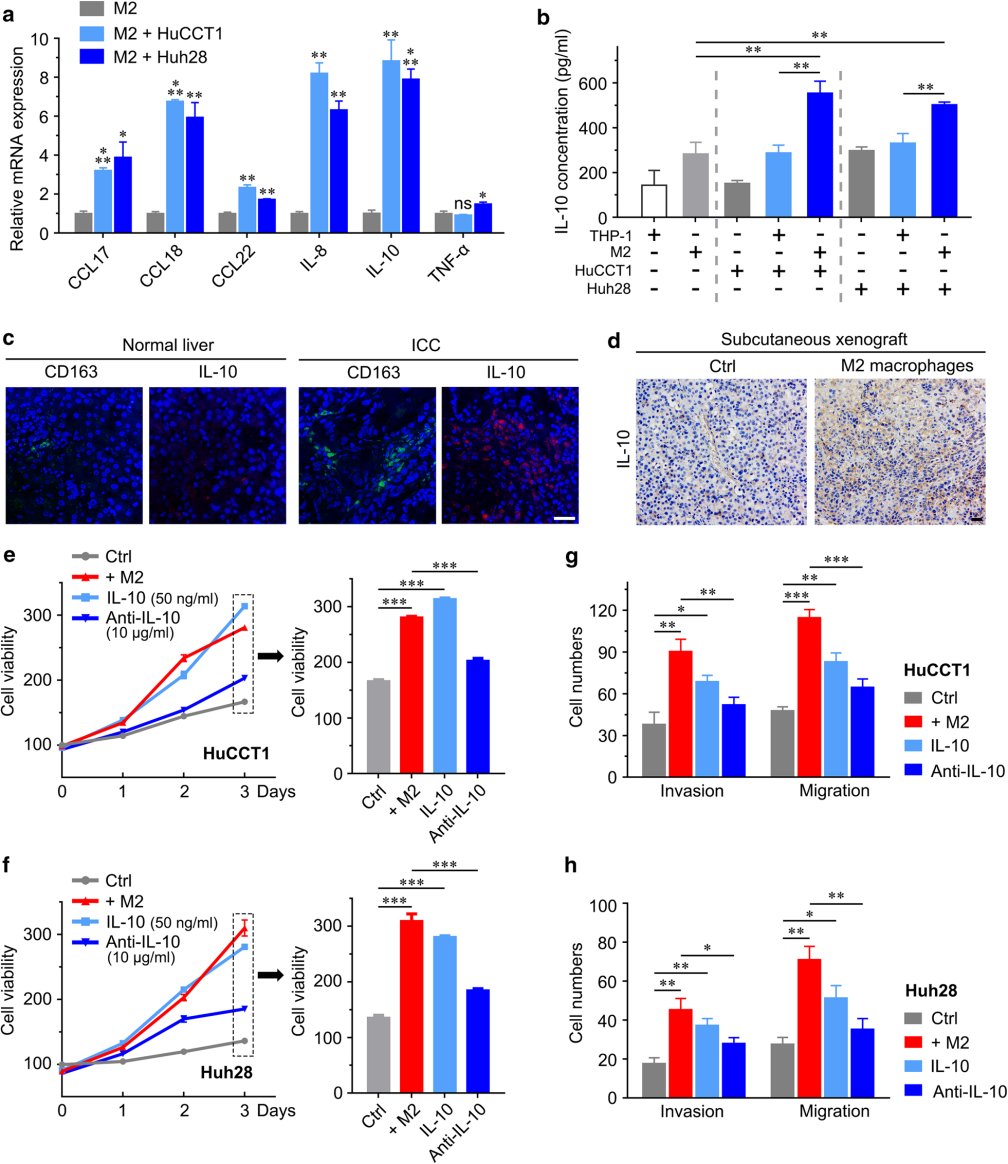
IL-10 is responsible for the pro-proliferation and migration effect of M2 macrophages. **a** Analysis of mRNA expression of secreted cytokines (CCL17, CCL18, CCL22, IL-8, IL-10, TNF-α, IL-6 and COX-2) in M2 macrophages before and after coculture with HuCCT1 cells by qRT-PCR. **b** Measurements of IL-10 levels in the supernatants of THP-1, M2, HuCCT1 or Huh28 cells, THP-1/ICC cells, and M2/ICC cells by ELISA. **c** Immunofluorescence image of a clinical peritumoral section labeled with antibodies against IL-10 (red) and CD163 (green). **d** Immunohistochemical detection of IL-10 in subcutaneous tumor sections. **e**, **f** CCK8 assays using IL-10-treated ICC cells and ICC cells cocultured with M2 macrophages and treated with IL-10 neutralizing antibody (IL-10 Ab). **g**, **h** Invasion and migration assays using IL-10-treated ICC cells and ICC cells cocultured with M2 macrophages and treated with IL-10 Ab. The data are presented as the mean ± SD; *p < 0.05, **p < 0.01, ***p < 0.001. Bar = 1 mm

M2 macrophages are known to secrete various kinds of cytokines to mediate their oncogenic effects [22, 23], which accounts for the previously described M2 macrophages abundantly present in the tumor peripheral area and their protumorigenic activities in ICC. Therefore, we assessed the expression of six secreted cytokines in M2 macrophages after coculture with HuCCT1 and Huh28 cell. Among the cytokines tested, IL-10 was highly expressed (*p* < 0.01, Fig. 5a). Interleukin-10 (IL-10) is one of the cytokines released by M2 macrophages and has been subsequently found to play multiple roles in cancer development. ELISA confirmed a significant increase in IL-10 in M2/HuCCT1 or Huh28 coculture supernatants (553.5 pg/ml, 494.8 pg/ml) compared to HuCCT1 (150.9 pg/ml), Huh28 (297.07 pg/ml), THP-1/HuCCT1 or Huh28 (286.7 pg/ml, 330.63 pg/ml), THP-1 (142.9 pg/ml) and M2 macrophage (281.9 pg/ml) supernatants (Fig. 5b), indicating that IL-10 was involved in the ICC induction effects. Subsequently, we further confirmed that M2 macrophages (CD163 +) were enriched in human ICC tissues and secreted IL-10 to exert their tumor-promoting effects (Fig. 5c). We found more IL-10-positive cells in subcutaneous xenografts from the Huh28/M2 macrophage co-injected group than in the control group (Fig. 5d).

Based on these observations, we added recombinant human IL-10 and its neutralizing antibody (IL-10 Ab) into the M2/ICC cell coculture system to determine whether IL-10 is associated with the proliferation and invasiveness of ICC cells. We found that IL-10 significantly promoted ICC cell proliferation, invasion and migration. Similarly, the IL-10 Ab partially blocked M2 macrophage-induced proliferation, invasion and migration of ICC cells (Fig. 5e–h).

These data indicated that IL-10 secreted from M2 macrophages plays a promoting role in the effects on proliferation and invasiveness in ICC.

### M2 macrophage-mediated enhancement of ICC malignancy through the STAT3 signaling pathway

**Fig. 6:**
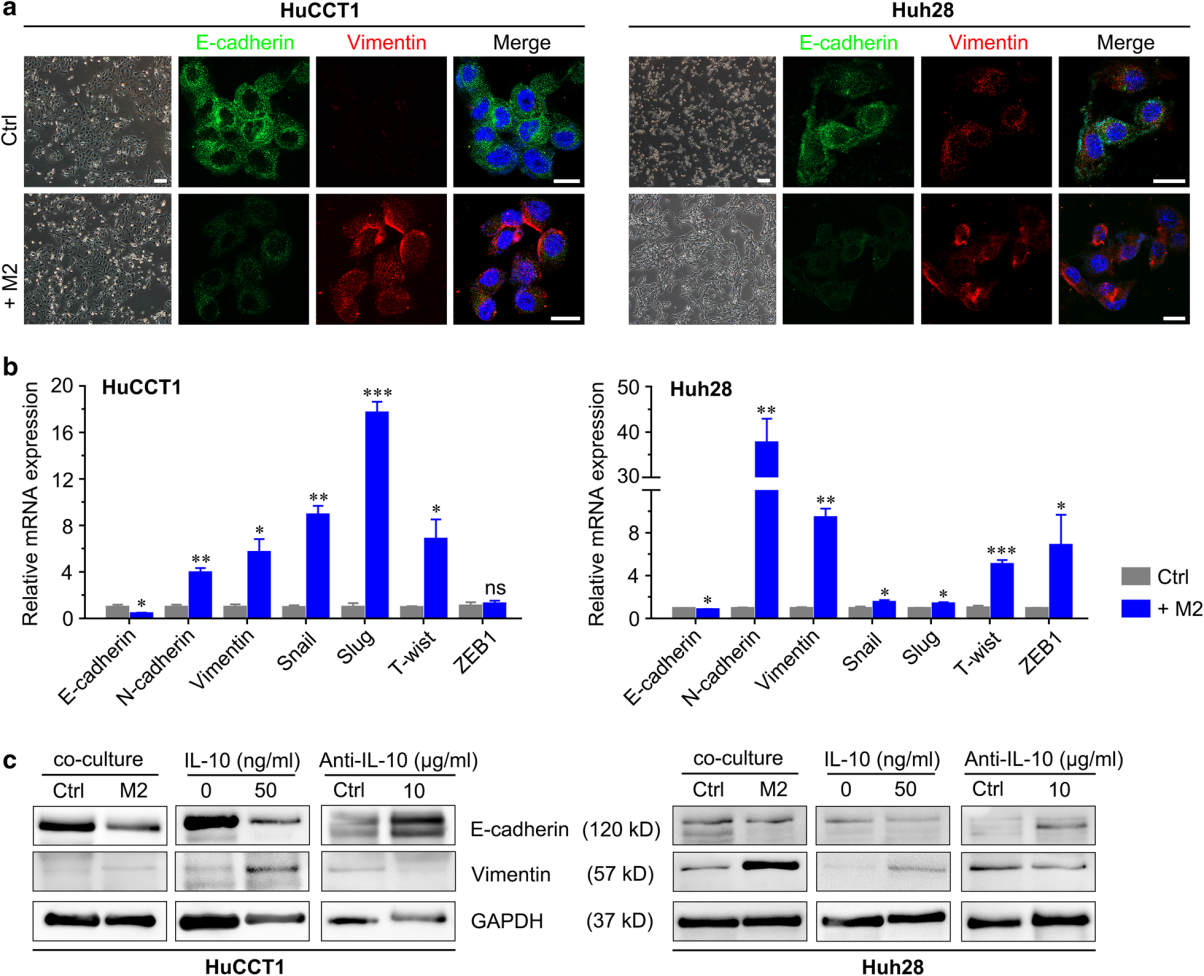
THP-1-derived M2 macrophages promote EMT in ICC cells. **a** Morphological changes during EMT of HuCCT1 and Huh28 cells after coculture with M2 macrophages. IF staining for EMT markers in HuCCT1 and Huh28 cells cocultured with THP-1-derived M2 macrophages. Green and red represent E-cadherin and vimentin marker staining, respectively, and the blue signal represents DAPI-stained nuclei. Bar = 1 mm. **b** qRT-PCR analyses of E-cadherin, N-cadherin, vimentin, Snail, Slug, Twist and ZEB1 expression in ICC cells cocultured with THP-1-derived M2 macrophages. **c** Western blotting analyses of E-cadherin and vimentin expression in ICC cells cocultured with M2 macrophages treated with IL-10 and its neutralizing antibody. The data are presented as the mean ± SD; *p < 0.05, **p < 0.01, ***p < 0.001. Bar = 1 mm

It has been reported that M2 macrophages can promote EMT in tumors [24, 25], a process that underlies metastasis in various cancers [13, 18]. Therefore, we investigated whether coculture of ICC cells with M2 macrophages affected the expression of EMT markers in ICC cells. After 3 days of coculture with M2 macrophages, the morphology of ICC cells, which were originally round-shaped, changed to fusiform- or spindle-shaped morphology. This process is highly consistent with EMT (Fig. 6a). The results of qRT-PCR and Western blotting showed that the expression of the epithelial phenotype marker E-cadherin was decreased, while that of the mesenchymal markers vimentin and N-cadherin were increased in ICC cells cocultured with M2 macrophages compared with the control (Fig. 6b, c). The immunofluorescence results in ICC cells confirmed these changes (Fig. 6a). In addition, we found that the expression of EMT-inducing transcription factors in ICC cells, such as Snail, Slug, Twist and ZEB1, was significantly elevated after coculture with M2 macrophages, as demonstrated by qRT-PCR (Fig. 6b). These results indicated that M2 macrophages may promote the migration and invasion of ICC cells through EMT.

**Fig. 7:**
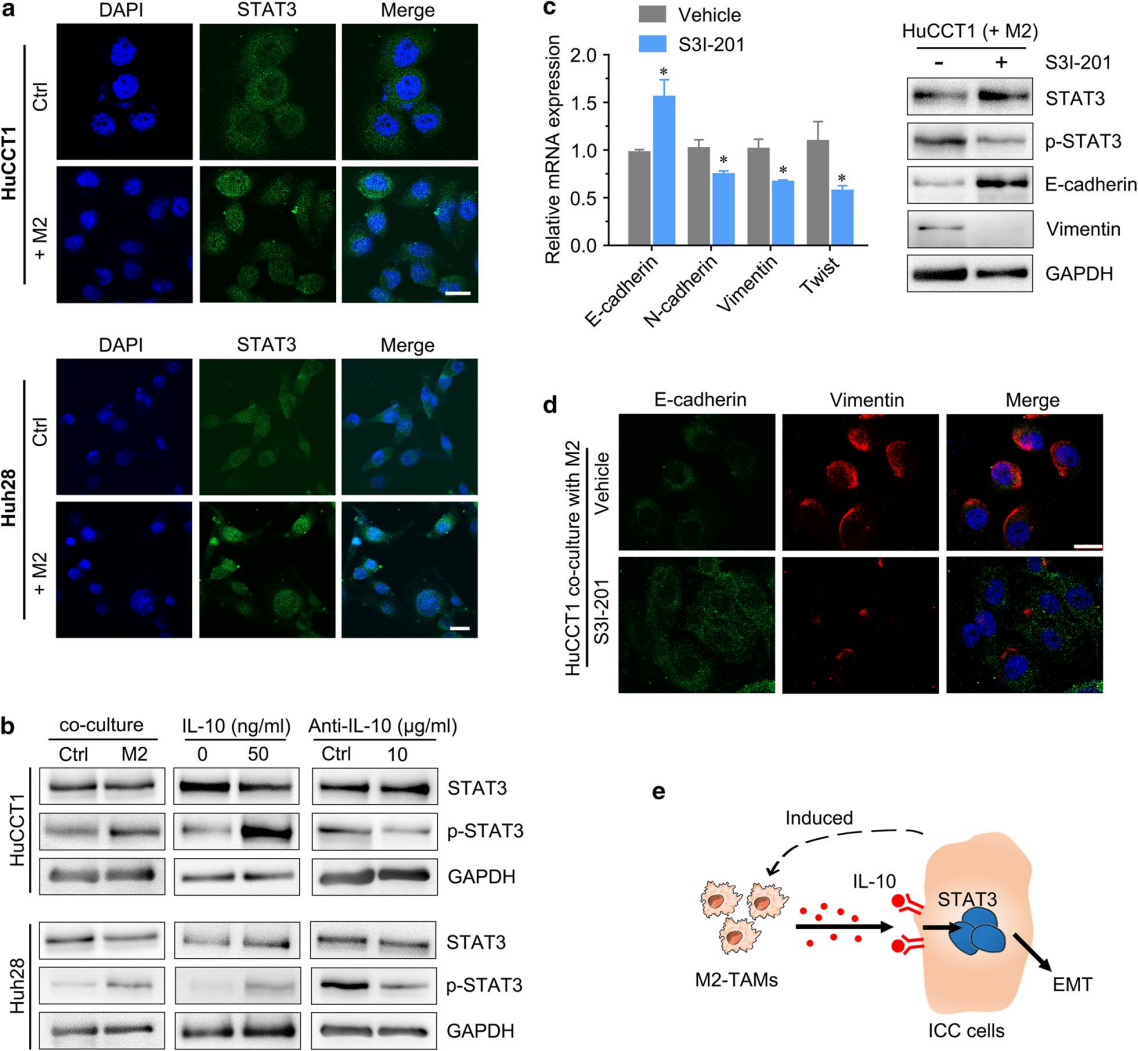
STAT3 signaling is critical for M2 macrophage-induced EMT in ICC cells. **a** IF staining for STAT3 in HuCCT1 and Huh28 cells cocultured with THP-1-derived M2 macrophages. The green color represents STAT3 staining, and the blue signal represents DAPI-stained nuclei. **b** Western blotting analyses of STAT3 and p-STAT3 expression in ICC cells cocultured with M2 macrophages treated with IL-10 and its neutralizing antibody. **c** IF staining for EMT markers in HuCCT1 cells cocultured with M2 macrophages after treatment with STAT3 inhibitor (S3I-201). **d** The left panel shows EMT marker expression in HuCCT1 cells after treatment with S3I-201, as determined by qRT-PCR, and the right panel shows the changes in p-STAT3 and EMT markers (E-cadherin and vimentin). **e** A schematic model describing the mechanism investigated in the present study in which ICC induces M2-polarized tumor-associated macrophages, and IL-10 secreted by M2 macrophages promotes tumor growth, invasiveness and EMT via STAT3 signaling. The data are presented as the mean ± SD; *p < 0.05. Bar = 1 mm

Signal transducer and activator of transcription 3 (STAT3) signaling has been considered a key inducer of ICC tumorigenesis [26], and IL-10 can activate STAT3 signaling in tumors. To investigate whether IL-10 promotes EMT in ICC cells by activating STAT3, we attempted to evaluate the activation status of the STAT3 signaling pathway, one of the most commonly involved pathways in the EMT process [27, 28]. We found that ICC cells cocultured with M2 macrophages significantly enhanced STAT3 phosphorylation, and the p-STAT3 levels were increased when rhIL-10 was added to the coculture system (Fig. 7a, b). Furthermore, IL-10 Ab in the coculture system partly reduced p-STAT3 levels (Fig. 7b). The STAT3 inhibitor S3I-201 was used to further substantiate the finding that ICC cells cocultured with M2 macrophages induce EMT by activating STAT3 signaling. We found that the expression of p-STAT3 and EMT markers in ICC cells cocultured with M2 macrophages were partly reversed by S3I-201 (Fig. 7c, d).

These results indicated that IL-10 secreted by M2 macrophages may promote the migration, invasion and EMT of ICC cells through the STAT3 signaling pathway.

## Discussion

ICC is a highly malignant type of adenocarcinoma that originates from the epithelium of the bile duct [29]. Currently, surgical resection is the only effective but unsatisfactory treatment for ICC, and the risk of recurrence of ICC is still high, with a 5 year survival rate of only 20–40% after surgery [30, 31]. Therefore, an important research direction is to explore the tumorigenic mechanisms and find effective molecular targets for ICC.

Tumor-associated macrophages are key orchestrators of the tumor microenvironment and play an important role in modulating tumor development and progression [8, 12]. Specifically, macrophages can be differentiated into two subtypes: M1 macrophages and M2 macrophages. M1 macrophages are able to kill tumor cells, while M2 macrophages act as “protumoral macrophages” and promote the initiation and metastasis of tumor cells and subsequent tumor progression [20, 32, 33]. Although it has been reported that M2 macrophages may promote EMT partially by increasing the secretion of cytokines and activating the AKT3/PRAS40 signaling pathway in ICC in vitro [34], the molecular mechanisms of ICC malignancy are not completely clear, and with the lack of clinical evidence, very little is known regarding their role in ICC in vivo. Our study revealed the interactions between ICC cells and M2 macrophages and how they contribute to the growth and invasiveness of ICC in vitro and in vivo. We first explored the role of M2 macrophages in ICC murine models and human samples and found that majority of macrophages located in the peritumoral regions exhibited the M2 phenotype, and the number was significantly higher than that in normal liver tissue.

Most functions of macrophages are achieved through the secretion of signaling inducers or regulatory cytokines [22, 23]. M2 macrophages promote tumor malignant characteristics directly or indirectly through the paracrine activation of various cytokines and chemokines [35]. IL-6 has been confirmed to activate STAT3 pathway. In present study, we assessed the expression of several secreted cytokines in M2 macrophages after co-culture with HuCCT1 or Huh28 cells. We found the upregulated level of IL-6, IL-8, IL-10, IL-13 and TGF-β, and IL-10 was the most significant increased cytokines. IL-10 is an important inflammatory cytokine and has been shown to play a regulatory role in immune and inflammatory responses [36–38]. Moreover, IL-10 was reported to play important role in cancer development and to strongly induce the phosphorylation of STAT3 in cancer cells [39]. Collectively, we focused the role of IL-10 in the crosstalk between M2 macrophages and intrahepatic cholangiocarcinoma cells. In this study, we validated that IL-10 could promote ICC malignancy by promoting EMT.

We successfully induced M2 macrophages from THP-1 cells in vitro and confirmed subtype-related marker expression (CD206, CD163 and Arg-1) by qRT-PCR. To simulate the tumor microenvironment while preventing direct contact between cells, we used a transwell coculture system of M2 macrophages and ICC cells. After coculture, we found that the morphology of ICC cells (both HuCCT1 and Huh28) were changed along with the aberrant expression of EMT markers. In addition, IL-10 levels in cell coculture supernatants were significantly increased compared with those in untreated THP-1 cells. It has been reported that IL-10 can regulate proliferation, invasiveness, and EMT by activating STAT3 signaling in other malignant tumors.

Therefore, to determine whether IL-10 secreted by M2 macrophages promotes the progression and development of ICC by activating STAT3, we added recombinant human IL-10, IL-10 neutralizing antibody or a STAT3 inhibitor to the M2/ICC cell coculture system in vitro. We found that IL-10 significantly promoted ICC cell proliferation, invasiveness and EMT by activating STAT3, and these effects exerted by M2 macrophages could be partly eliminated after blocking IL-10/STAT3. We used a subcutaneous co-transplantation tumor model in nude mice to visually observe the tumor growth effect of M2 macrophages in vivo*.* ICC cells co-cultured with M2 macrophages or THP-1 cells and then together used to build the in vivo invasive assay, such as the peritoneal metastasis or long-distance lung metastasis, seem not very suitable and not enough to verify the capacity of M2-macrophages induced by intrahepatic cholangiocarcinoma in promoting tumor invasion [40]. Therefore, we verify the changes in the expression of EMT markers in subcutaneously co-transplantation tumor mouse model. E-cadherin was decreased while vimentin and IL-10 were simultaneously increased in the experimental group, as demonstrated by immunohistochemistry, suggesting that M2-TAMs modulate malignant properties via IL-10/STAT3 in ICC cells and could block the effect of the signaling molecule-specific repressor.

## Conclusion

In conclusion, monocytes are recruited to the peritumoral region in ICC and are polarized towards M2-TAMs. IL-10 secreted by M2-TAMs promotes malignant characteristics and EMT in ICC through STAT3 signaling. Blockade of IL-10/STAT3 signaling partly rescued the protumor effects of M2 macrophages on ICC. Therefore, it might be a novel therapeutic strategy for ICC patients (Fig. 7e).

## Supplementary Information

**Additional file 1:** **Table S1. **List of antibodies used in this study.

**Additional file 2: Table S2.** Real-time polymerase chain reaction primers.

## Data Availability

All data generated or analyzed during this study are included in this published article and its supplementary information files.

## Abbreviations

ICC: Intrahepatic cholangiocarcinoma
M2-TAMs: M2-polarized tumor-associated macrophages
EMT: Epithelial–mesenchymal transition
DEN: Diethylnitrosamine
LMBDL: Left median bile duct ligation
CCK-8: Cell counting kit-8
FBS: Fetal bovine serum
IHC: Immunohistochemistry
qRT-PCR: Real-Time Quantitative PCR
STAT3: Signal transducer and activator of transcription 3
