# Supplementary material for: Intrahepatic cholangiocarcinoma induced M2-polarized tumor-associated macrophages facilitate tumor growth and invasiveness
Source: Cancer Cell Int. 2020 Dec 7;20:586. doi: 10.1186/s12935-020-01687-w (PMC7720384; doi:10.1186/s12935-020-01687-w)
Supplement: Supplementary file 1 — Additional file 1: Table S1. List of antibodies used in this study. [file 12935_2020_1687_MOESM1_ESM.docx]

**Supplementary Table1.**  List of antibodies used in this study.

| **Primary antibody** | **Experiment** | | **Source** | **Dilution** |
| --- | --- | --- | --- | --- |
| CD163 | IHC/IF | Abcam (ab182422) | | 1:100 |
| CD206 | IHC | Abcam (ab64693) | | 1:1000 |
| CK19 | IHC | Abcam (ab52625, ab9377) | | 1:100 |
| E-cadherin | IHC/IF | Cell Signaling Technology (3195) | | 1:100 |
| F4/80 | IHC/IF | Abcam (ab6640) | | 1:100 |
| IL-10 | IHC/IF | Abcam (ab34843) | | 1:400/1:100 |
| iNOS | IHC/IF | Abcam (ab15323) | | 1:100 |
| Ki-67 | IHC | Abcam (ab16667) | | 1:100 |
| STAT3 | IF | Cell Signaling Technology (9139) | | 1:100 |
| p-STAT3 | IHC | Cell Signaling Technology (9145) | | 1:200 |
| Vimentin | IHC/IF | Abcam (ab92547) | | 1:200/1:100 |
| E-cadherin | Western blotting | BD (610682) | | 1:1000 |
| GAPDH | Western blotting | Cell Signaling Technology (5174) | | 1:1000 |
| STAT3 | Western blotting | Cell Signaling Technology (9139S) | | 1:1000 |
| p-STAT3 | Western blotting | Cell Signaling Technology (9145S) | | 1:1000 |
| Vimentin | Western blotting | Cell Signaling Technology (5241S) | | 1:1000 |

**Abbreviations**: IHC, immunohistochemistry; IF, immunofluorescence.
