# Supplementary material for: Intrahepatic cholangiocarcinoma induced M2-polarized tumor-associated macrophages facilitate tumor growth and invasiveness
Source: Cancer Cell Int. 2020 Dec 7;20:586. doi: 10.1186/s12935-020-01687-w (PMC7720384; doi:10.1186/s12935-020-01687-w)
Supplement: Supplementary file 2 — Additional file 2: Table S2. Real-time polymerase chain reaction primers. [file 12935_2020_1687_MOESM2_ESM.docx]

**Supplementary Table2.** Real-time polymerase chain reaction primers.

| Gene | Forward Primer (5’ to 3’) | Reverse Primer (5’ to 3’) |
| --- | --- | --- |
| *Arg1*  *CCL17*  *CCL18*  *CCL22*  *CD163*  *CD206*  *COX2*  *E-cadherin*  *GAPDH*  *IL-6*  *IL-8*  *IL-10*  *iNOS*  *N-cadherin*  *Slug*  *Snail*  *Twist*  *Vimentin*  *ZEB1* | GTGGAAACTTGCATGGACAAC  CGGACCCCAACAACAAGAGA  GTTGACTATTCTGAAACCAGCCC  AGGACAGAGCATGGATCGCCTACAGA  CCAGAAGGAACTTGTAGCCACAG  AGCCAACACCAGCTCCTCAAGA  CTGGCGCTCAGCCATACAG  TCGACACCCGATTCAAAGTGG  TGCACCACCAACTGCTTAGC  TTCTCCACAAGCGCCTTC  TTTTGCCAAGGAGTGCTAAAGA  GACTTTAAGGGTTACCTGGGTTG  TTCAGTATCACAACCTCAGCAAG  GCGCGTGAAGGTTTGCCAGTG  TTCGGACCCACACATTACCT  AAGGATCTCCAGGCTCGAAAG  GGAGTCCGCAGTCTTACGAG  TGGCCGACGCCATCAACACC  TACAGAACCCAACTTGAACGTCACA | AATCCTGGCACATCGGGAATC  ACTGTGGCTCTTCTTCGTCC  GTCGCTGATGTATTTCTGGACCC  TAATGGCAGGGAGGTAGGGCTCCTGA  CAGGCACCAAGCGTTTTGAGCTA  CAAAACGCTCGCGCATTGTCCA  CGCACTTATACTGGTCAAATCCC  TTCCAGAAACGGAGGCCTGAT  GGCATGGACTGTGGTCATGAG  AGCAGGCAACACCAGGAG  AACCCTCTGCACCCAGTTTTC  TCACATGCGCCTTGATGTCTG  TGGACCTGCAAGTTAAAATCCC  CCGGCGTTTCATCCATACCACAA  GCAGTGAGGGCAAGAAAAAG  GCTTCGGATGTGCATCTTGA  TCTGGAGGACCTGGTAGAGG  CACCTCGACGCGGGCTTTGT  GATTACACCCAGACTGCGTCACA |
